# A Water Level Measurement Approach Based on YOLOv5s

**DOI:** 10.3390/s22103714

**Published:** 2022-05-13

**Authors:** Guangchao Qiao, Mingxiang Yang, Hao Wang

**Affiliations:** 1 College of New Energy and Environment, Jilin University, No. 2519, Jiefang Road, Chaoyang District, Changchun 130000, China; qiaogc21@mails.jlu.edu.cn; 2 China Institute of Water Resources and Hydropower Research, No. 1 Yuyuantan South Road, Haidian District, Beijing 100038, China; wanghao@iwhr.com; 3 State Key Laboratory of Simulation and Regulation of Water Cycle in River Basin, No. 1 Yuyuantan South Road, Haidian District, Beijing 100038, China

**Keywords:** water level measurement, machine vision, image processing, object detection

## Abstract

Existing water gauge reading approaches based on image analysis have problems such as poor scene adaptability and weak robustness. Here, we proposed a novel water level measurement method based on deep learning (YOLOv5s, convolutional neural network) to overcome these problems. The proposed method uses the YOLOv5s to extract the water gauge area and all scale character areas in the original video image, uses image processing technology to identify the position of the water surface line, and then calculates the actual water level elevation. The proposed method is validated with a video monitoring station on a river in Beijing, and the results show that the systematic error of the proposed method is only 7.7 mm, the error is within 1 cm/the error is between 1 cm and 3 cm, and the proportion of the number of images is 95%/5% (daylight), 98%/2% (infrared lighting at night), 97%/2% (strong light), 45%/44% (transparent water body), 91%/9% (rainfall), and 90%/10% (water gauge is slightly dirty). The results demonstrate that the proposed method shows good performance in different scenes, and its effectiveness has been confirmed. At the same time, it has a strong robustness and provides a certain reference for the application of deep learning in the field of hydrological monitoring.

## 1. Introduction

Water level monitoring is an important part of hydrological monitoring; continuous and reliable water level monitoring is of great significance for water safety guarantee, water resource management, and water environment and water ecology protection. At present, water level measurement approaches include manual reading, automatic water level measurement, and optical fiber-based liquid level measurement technology [1]. Manual reading is used to observe the water gauge with the naked eye; however, the reading frequency is low and it has a certain degree of subjectivity. The readings are greatly affected by the observation angle, and the uncertainty is greater in harsh environments. The methods of obtaining water level data through automatic water level gauge benefit from the development of electronic technology-based sensor technology. The existing automatic water level gauges include float type, pressure type, ultrasonic type, radar type, capacitive type, etc. Paul [2] measured the water level with the Garmin radar sensor. When the tilt angle is not greater than 40°, the sensor has high accuracy; when the measurement distance is less than 10 m, the accuracy is less than 1 cm, and the maximum detectable range is 30 m to 35 m. Li [3] collected water level data through an ultra-small pressure sensor, with an error of 1–2 cm. These water level gauges have their own advantages, but the disadvantages are also more obvious. The current sensors for measuring water level have problems such as short product life, low measurement accuracy and unstable performance. They work in a complex manner and have high maintenance costs. Their accuracy is easily affected by the environment, and they cannot achieve 24-h monitoring. At present, many water level monitoring sites have built video surveillance system and are equipped with upright water gauge. They only use the video real-time viewing, recording and playback functions and have not fully utilized the role of video remote monitoring. Although manual observation of water level data is still required, it consumes manpower and material resources, and it is difficult to analyze the water level data obtained.

With the maturity of deep learning and image processing theory and technology, artificial intelligence has shown unprecedented advantages in many fields, such as medical imaging [4], autonomous driving [5], face recognition [6], smart transportation, and so on, greatly improving people’s production efficiency. Some scholars have found that the waterline is determined by the horizontal projection of grayscale images and edge images, which effectively overcomes the shortcomings of dark light, glare, shadow, and artificial night lighting in complex scenes, and its accuracy reaches 1 cm [7]. Eltner [8] automatically extracted the water level through image processing. The research results show that it performs well in most simple scenes, with an average error of less than 6 mm, but this method may fail when the image has a complex background. Lo [9] captured an image from the scene at a rate of one per minute to monitor the water level of the city river. It determines the water level by the water gauge on the bridge pier. In addition, the author mentioned the use of virtual markers when there is no water gauge in the monitoring area. The existing approaches are generally not universal, and they do not solve the problem of scene adaptability. These approaches are effective only under certain conditions, and there are large limitations.

Aiming at preliminarily solving problems in the existing research, this paper proposed a more versatile and robust approach. The proposed method makes full use of deep learning, locates the water gauge in the video image through the object detection algorithm, performs preprocessing such as grayscale, binarization, and image denoising, and projects the processed image in the horizontal direction to determine the water surface line coordinates, after which we use the object detection algorithm to locate all the scale characters on the water gauge, and obtain the final water level data through calculation and conversion according to the actual value represented by the bottom scale character.

In the following sections, we describe our method in more detail. In Section 2, we give an overview of the algorithms and data used in the paper. In Section 3, we explain our proposed approach in more detail. Detailed experimental results and a discussion are given in Section 4. Section 5 draws the conclusion of this paper.

## 2. Algorithms and Data

### 2.1. Algorithms

#### 2.1.1. YOLOv5s

In 2014, the R-CNN algorithm [10] proposed by Girshick defeated the peak of the traditional object detection algorithm DPM [11] by an absolute advantage on the PASCAL VOC dataset. It reached a new milestone for the application of deep learning in the field of object detection. Since then, the research of deep learning algorithms in the field of object detection has occupied an absolute dominant position, and this has continued to this day. Object detection algorithms based on deep learning are usually divided into two kinds: one-stage object detector and two-stage object detector. The most representative two-stage object detector is the R-CNN series, which includes Fast R-CNN [12], Faster R-CNN [13], R-FCN [14], and Libra R-CNN [15]. As for one-stage object detectors, the most representative models are YOLO [16,17,18,19], SSD [20], and RetinaNet [21]. The two-stage algorithm starting from Faster R-CNN has achieved end-to-end training, but there is still a big gap with the real-time requirements of practical application scenarios, while YOLO truly achieves real-time object detection. YOLO gives all the detection results at once based on the entire input image. After several years of update iterations, in 2020, Ultralytics LLC proposed YOLOv5.

**Figure 1:**
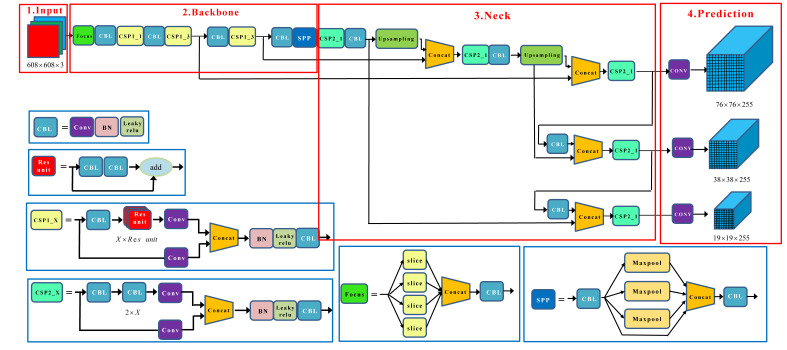
The structure of YOLOv5s.

YOLOv5 integrates many excellent academic achievements in recent years on the basis of YOLOv4. It has higher detection accuracy and speed while being more flexible in network deployment, making it an ideal candidate for real-time condition and mobile deployment environment. According to the size of the model, YOLOv5 includes YOLOv5s, YOLOv5m, YOLOv5l, and YOLOv5x. The weight, width, and depth of these four versions of the model are increased in turn, and they are all composed of input, backbone, neck, and head. Among them, the part of input uses mosaic data augmentation, auto learning bounding box anchors, and image scaling to preprocess the image. The backbone part uses the focus downsampling, improved BottleneckCSP, and SPP (Spatial Pyramid Pooling) structures to extract feature information of image. The neck part combines the feature pyramid structure (Feature Pyramid Networks, FPN) with the path aggregation network structure (Path Aggregation Network, PAN) [22], which realizes the transfer of feature information of objects of different sizes and solves the problem of multi-scale object detection. The head part uses three loss functions to calculate classification, localization, and confidence loss, respectively, and improves the accuracy of network through NMS (Non-Maximum Suppression). Taking YOLOv5s as an example, its structure is shown in Figure 1.

(1) Input

In the part of input, mosaic data augmentation is used in the training phase of the model, and the four images are stitched according to random scaling, random cropping and random arrangement. The number of small objects in the dataset increases, thereby improving the detection ability of small objects; YOLOv5 can start the adaptive anchor box calculation according to the parameters, and automatically learn to generate anchors according to the training data. It improves the previous YOLOv3 and YOLOv4 using the clustering method to manually generate anchors into an automatically generated method; we think this innovation point is less significant. Adaptive scaling allows to scale the original image to a uniform standard size, and then input it into the detection network (when scaling, keep the original scale of the image unchanged, and use the least padding).

(2) Backbone

As shown in Figure 1, in backbone, the focus structure is a slicing operation on the feature map. The input image is divided into four parts according to two times downsampling, and the 12-dimensional feature map is obtained by splicing in the channel dimension. The feature information is further extracted through a 3 × 3 composite convolution module to generate a 32-dimensional feature map. Less information is lost after focus downsampling, and FLOPS (floating point operations per second) is reduced through reshape, which improves the detection speed; YOLOv5 adopts CSPNet to extract rich informative features from input image and CSPNet integrates the gradient changes into the feature map, reducing the parameter amount and FLOPS value of the model. This not only ensures the inference speed and accuracy, but also reduces the size of model. It solves the gradient information duplication problem of network optimization in other large convolutional neural network backbone frameworks; SPP uses a combined three multi-scale max-pooling layers, which can improve the receptive field almost without slowing down the speed, which helps to solve the alignment problem of anchors and feature layers.

(3) Neck

In the neck part of Figure 1, FPN improves the detection effect of small objects by fusing high- and low-layer features. At the same time, PAN adds a bottom-up connection path on the basis of FPN, so that the location information of the bottom layer can be better transmitted to the top layer. The combination of FPN and PAN can perform feature aggregation for different detection layers from different backbone layers. This structure can further improve the detection performance of dense objects, and strengthen the ability of the network to fuse the features of objects with different scales.

(4) Head

In the part of head in Figure 1, the GIOU (Generalized Intersection over Union) is used as the loss function of the bounding box. In the post-processing process of object detection, NMS is used to filter multi-object boxes, which enhances the detection ability of multiple objects and occluded objects.

#### 2.1.2. Image Classification

Image classification is a common task in the field of computer vision, that is, given an input image, a certain classification algorithm is used to determine the category of the image. The main process of image classification includes image preprocessing, feature extraction, and classifier design. In image classification tasks, good feature expression plays a vital role in improving the classification accuracy of images. Deep learning relies on neural networks, and with its powerful feature extraction capabilities, it has been widely used in the field of computer vision.

At present, Convolutional Neural Networks (CNN) is a widely used artificial neural network. CNN is developed from the artificial neural network, and has many similarities with the artificial neural network. For example, the output of the front layer is the input of the back layer between the layers of the network, and the parameters are updated through the back propagation algorithm. At the same time, there are many differences between them. First, CNN is not fully connected, as CNN is a locally connected network. Compared with the typical BP fully connected neural network, it has the characteristics of local connectivity and weight sharing. Secondly, the CNN model contains more network layers, and some complex network models have as many as 100 layers. Finally, the network structure of CNN includes: the input layer, convolutional layer, activation function, pooling layer, and fully connected layer.

### 2.2. Data

#### 2.2.1. Water Gauge Image Data

**Figure 2:**
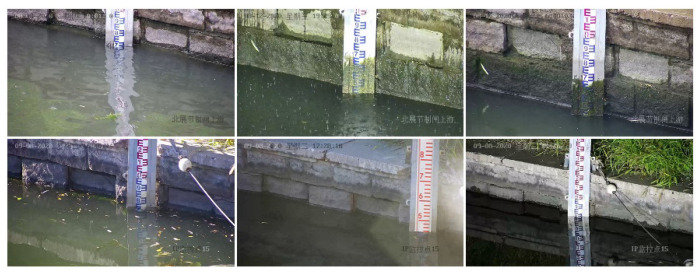
Classic image samples.

The water gauge image data are collected through two ways of Internet downloading and on-site shooting, totaling 5000 sheets. Among them, the web crawling method uses crawler technology to download images related to the water gauge to the local area, and screens out sample images with better quality through manual observation, although its resolution varies; on-site shooting mode uses video monitoring equipment to collect real business demand scene images of a hydrological monitoring sites in Beijing, capturing high-definition images of 1920 × 1080 pixels, where each video image contains a water gauge object. In order to make the collected samples more general and enable the network to fully learn and use these samples, the collected samples come from different scenes as much as possible. For example, the water gauge has problems such as different viewing angles, different lighting, and different weather. For issues such as viewing angle and illumination, the camera angle is adjusted multiple times when images are collected and the water gauge is taken at different time periods to enrich the network to obtain the representation characteristics. The actual field environment is relatively complex, with many scene factors, such as water surface wave, water surface reflection, transparent water body, backlighting, night fill light overexposure, dirty water gauge, and floating objects on the water surface, which bring huge challenges to object detection. Figure 2 shows part of the collected samples (In each sub-picture in the figure, the text in the upper left corner is the date the sample was collected, and the text in the lower right corner is the name of the video surveil-lance site).

#### 2.2.2. SVHN

**Figure 3:**
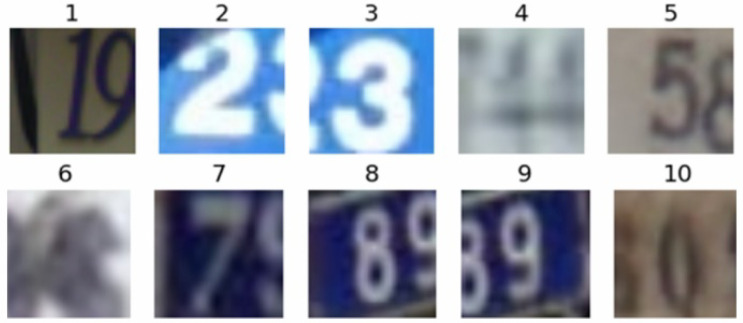
The processed images in SVHN.

The SVHN is a Google Street View House Number dataset, which contains three subsets: a training set, test set, and validation set. Among them, the training set contains 73,257 digital images, the test set contains 26,032 digital images, and the validation set consists of 531,131 digital images. The dataset is divided into 10 categories, and each category represents a number. For example, the category label of the character “1” is 1, and so on, and the label of the character “0” in the dataset is 10. This paper uses Formate2 format data; the original image is normalized to a 32 × 32 color image. In practical application, there is almost no need for preprocessing and format conversion, which is convenient for research algorithms in machine learning and object recognition. Some images in the dataset are shown in Figure 3.

## 3. Method Description

### 3.1. Water Gauge Detection Based on YOLOv5s

#### 3.1.1. Image Samples Annotation

**Figure 4:**
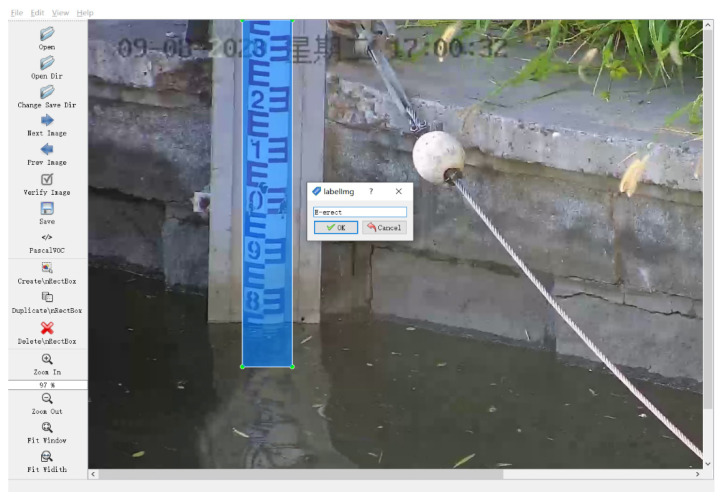
Sample annotation.

For the object detection task, after collecting the original images of the water gauge, we used the smallest bounding box to label all the water gauge objects in the images. This paper uses LabelImg to label the water gauge (E-erect) in the image and generate the corresponding xml format label file, as shown in Figure 4 (The text in the upper left corner of the picture is the date of sample collection).

#### 3.1.2. Water Gauge Location and Recognition

The image-based water level measurement method is to obtain the water gauge readings based on the image containing the water gauge. The water gauge is an important part of the realization of this method, and there are many factors that have a greater influence on the water gauge reading, such as the type of the water gauge and scene factors. There are many types of upright water gauges (with scale characters), e.g., E-shaped standard water gauge, scale line water gauge, etc. They are generally made of wood, enamel board, aluminum alloy, and 304 stainless steel, including flat and curved and so on, and the width of the water gauge is different, i.e., there are water gauges with widths of 8 cm, 15 cm, and 20 cm.

The primary task of the image-based water level measurement method is to locate the water gauge object in the video image and eliminate the interference of the complex background for subsequent processing. Existing water gauge location methods use image processing techniques such as grayscale, binarization, and edge detection to locate the water gauge in the image. These methods can show a good performance in simple and specific scenes, but the scene adaptability is poor. Once the scene is switched, these methods may fail. Therefore, this paper is based on the object detection algorithm YOLOv5s to automatically locate and recognize the water gauge in the image, and mark the detected water gauge object through a bounding box to obtain a set of bounding box prediction parameters.

### 3.2. Water Surface Line Recognition

#### 3.2.1. Image Preprocessing

Considering that the camera may be tilted, firstly, Hough transform is used to correct the tilt of the extracted RGB color water gauge image. Hough transform [23] is more robust and less affected by noise. Then, the water gauge image is grayed out and binarized, and the binarized water gauge image is obtained. In order to remove the noise in the binary image, Gaussian filtering is used to eliminate Gaussian noise [24], and median filtering [25] is used to eliminate impulse noise and salt and pepper noise. Since the value of each pixel of Gaussian filtering is obtained by the weighted average of itself and other pixel values in the neighborhood, the closer the distance to the object pixel, the greater the weight, the farther the distance, and the smaller the weight, and The edges of the binarized image become blurred. The protection of Gaussian filtering on high-frequency details is not obvious. Finally, it needs to be binarized again to sharpen the edge of the image.

#### 3.2.2. Binary Image Horizontal Projection

**Figure 5:**
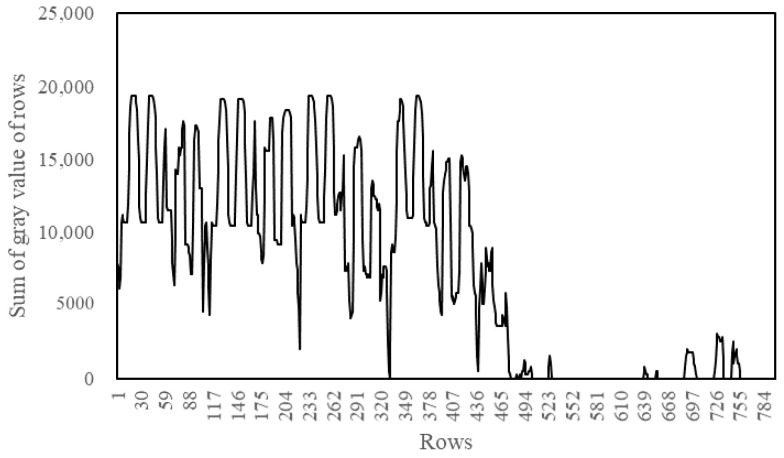
Horizontal projection curve.

In the obtained binary image, the water gauge is shown as a black and white texture area, while the water surface is shown as a black area with a significant difference, and the water surface line is the dividing line between the two parts. In order to detect the location of the water surface line, the binarized image is projected horizontally, that is, the pixel gray value in the binary image is accumulated in rows. Assume that the number of rows and columns of the binarized image are rows and cols, respectively, and the binary image is image. The calculation equation of the cumulative value of the pixel grayscale of a row is:

(1) $$\{\begin{array}{c} A(r,c)=image_{r,c}\\ SUM(r)=A(r,1)+A(r,2)+\cdots +A(r,cols) \end{array}$$

where r=1,2,3,⋯,rows,c=1,2,3,⋯,cols, A(r,c) is the pixel value of the *r*-th row and the *c*-th column of the binarized image and SUM(r) represent the accumulated value of the grayscale of each row of pixels, so that the horizontal projection curve of the binary image is obtained, as shown in Figure 5.

It can be seen from Figure 5 that the cumulative value of the 0–571 section of the curve presents regular fluctuations, corresponding to the water gauge area above the water surface; the section above 572 corresponds to the water surface area. There are sections with a cumulative value of 0 and many low cumulative values in this section. This is because there are small floating objects such as leaves on the water surface. There is a big difference between the horizontal projection curve of the water gauge and the water surface area, by setting an appropriate threshold δ, the strategy traverse search for SUM(row) from top to bottom. When SUM(row)<δ from a certain location for 10 consecutive times, then the position is taken as the row coordinate of the water surface line.

### 3.3. Character Location and Recognition of the Water Gauge

In the collected water gauge images, the scale character object to be inspected is small, and the number of scale characters of each category contained in the image sample is uneven. Existing deep learning-based object detection algorithms have a low detection accuracy in multi-category object detection. Therefore, in order to avoid problems such as unbalanced dataset classes and multi-class object detection [26], resulting in poor model performance. This paper classifies the scale characters of the ten categories from 0 to 9 into a category “num”, and respectively locate and recognize the scale characters in the water gauge image.

#### 3.3.1. Character Location

(1) Image sample cropping

According to the prediction parameters of the object bounding box of the water gauge obtained in Section 3.1, the object area of the water gauge is cut out, and the RGB color image of the water gauge area is obtained.

**Figure 6:**
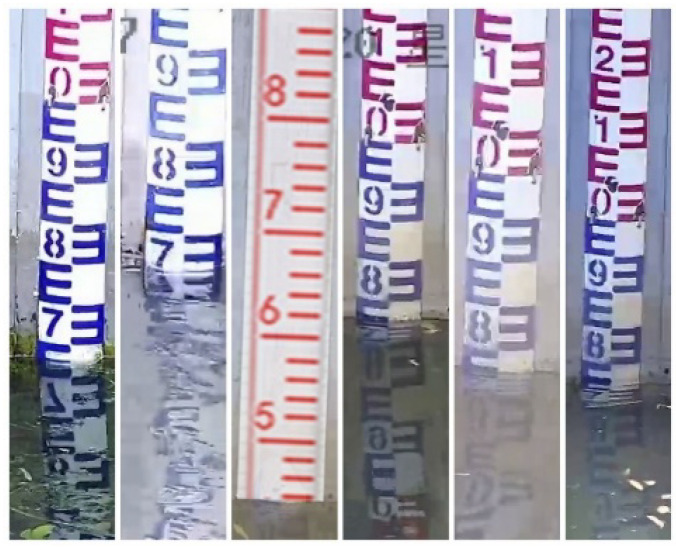
Sample cropping examples.

Character location is the object detection task. It is still necessary to make a dataset. The water gauge area in the original image is manually cut out to obtain the RGB color image of the water gauge area. There are 5000 sheets in total. Each sample image contains multiple scale characters, and the number of each category varies from 0 to 9. Some image samples are shown in the Figure 6.

(2) Image samples annotation

**Figure 7:**
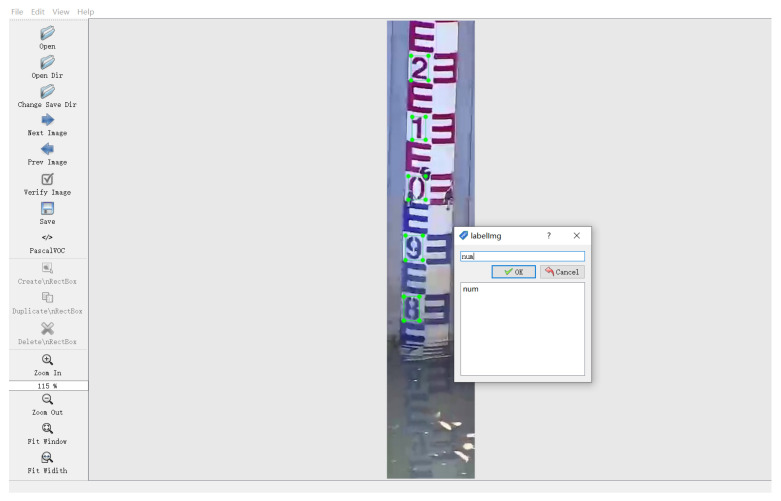
Labeling of scale character samples.

Similar to Section 3.1.1, LabelImg is used to label all scale characters in the water gauge images. An example of labeling is shown in Figure 7.

(3) Character location

Factors such as the complex lighting environment in the field and the small size of the scale characters have a greater impact on the location of the scale characters. Traditional character location methods have a poor locating accuracy and detection efficiency. This paper also uses the YOLOv5s model to locate the scale characters in the water gauge image, uses the bounding box to mark the detected character object, and obtains the bounding box prediction parameters of the detected character.

#### 3.3.2. Character Recognition

The character area at the bottom of the detected water gauge is cropped according to the bounding box prediction parameters to generate an RGB color image of the character, which is then input to the character recognition model to recognize the actual value represented. Recognizing scale characters in an image is essentially an image classification task. For the image classification task, convolutional neural networks are currently the best network structure and are widely used in many fields such as face recognition, automatic driving, and object detection [27]. This paper is based on the Keras deep learning framework to quickly design an image classification model to complete the scale character recognition task. The structure of the network is shown in Table 1.

As shown in Table 1, the character recognition model includes convolutional layers and fully connected layers. First, the convolutional layers are used to extract the features of the scale character image. Then, the convolutional layer is input to the fully connected layer after passing through the flatten layer. In order to prevent the model from overfitting, dropout [28] is used to discard some neurons. Finally, the softmax function is used to calculate the probability of each category of the input image, and the category with the highest probability is used as the category of the input image.

### 3.4. Determination of Water Level Data

**Figure 8:**
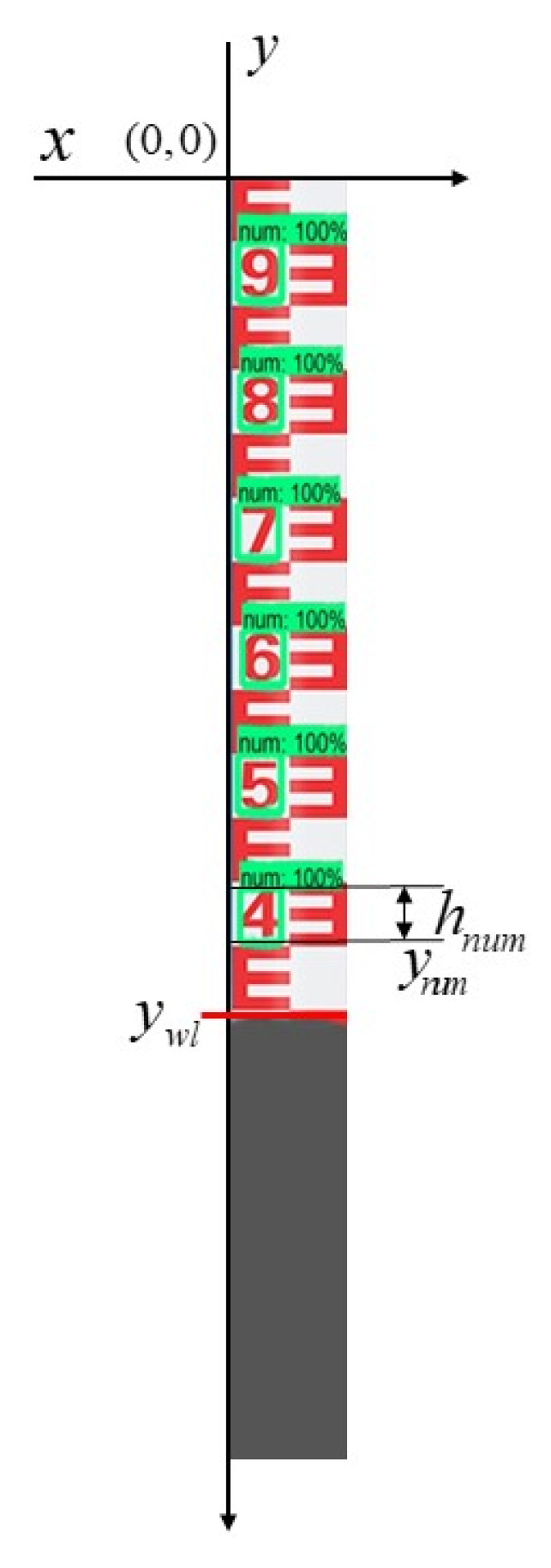
Determine water level data.

As shown in Figure 8, determining the actual height $h_{num}$ of the scale characters of the water gauge is a prerequisite for using the proposed method, and this parameter is relatively easy to obtain. We take the upper left corner of the water gauge image as the origin coordinate (0,0), the upper right corner direction is the positive *x*-axis direction (horizontal axis), and the lower left corner is the positive *y*-axis direction (vertical axis) to get the vertical axis coordinate value of the water surface line. The vertical distance between the water surface line and the origin coordinate is recorded as $y_{wl}$; starting from the position of the water surface line, look for the scale characters detected by the YOLOv5s in the opposite direction along the *y*-axis. When the first one is found, stop searching and record the vertical position as $y_{num}$ and the height as $h_{num}$. Then, recognize the actual value represented by the character through the scale character recognition model, and record the result as num. Finally, after a series of conversions and calculations, the final water level data WL are obtained.

The proposed method uses YOLOv5s to detect the position of the water gauge and the position of the scale characters in the water gauge area from the video image with complex background, which effectively eliminates the interference of the complex environment in the wild. This method is effective only when at least one scale character is detected, which greatly improves the accuracy of water level measurement.

It is worth noting that the core idea of this method is to convert the pixel distance between the detected lowermost scale character and the water surface line into the actual distance $y_{dis}$, the difference between the actual value is represented by the character num and $y_{dis}$ is the final water level elevation.

The proposed method can be applied in most complex scenes, and it is suitable for upright water gauges with scale characters. Not only that, the method can be directly applied after switching the camera perspective and the application site. Therefore, the proposed method has strong robustness.

## 4. Results and Discussion

### 4.1. Experimental Environment

The operating system of the experiments in this paper is Windows 10, the CPU is an Intel Xeon (Xeon) Gold 5218 @ 2.30 GHz (X2), the running memory is 64 GB, and the GPU is an NVIDIA Tesla T4 (15 GB/NVIDIA). The YOLOv5s is based on the Pytorch deep learning framework and it uses CUDA11.4.48 and cuDNN8.2.4 for GPU acceleration.

### 4.2. Water Gauge Detection Performance

In the training process, set the batch size to 16 and use the SGD (Stochastic Gradient Descent) optimizer. The model uses the warmup method to warm up the learning rate for the learning rate update. In the warmup stage, one-dimensional linear interpolation is used to update the learning rate for each iteration. After the warmup stage, the cosine annealing algorithm is used to update the learning rate, and the initial learning rate is 0.01. The number of iterations is set to 300.

**Figure 9:**
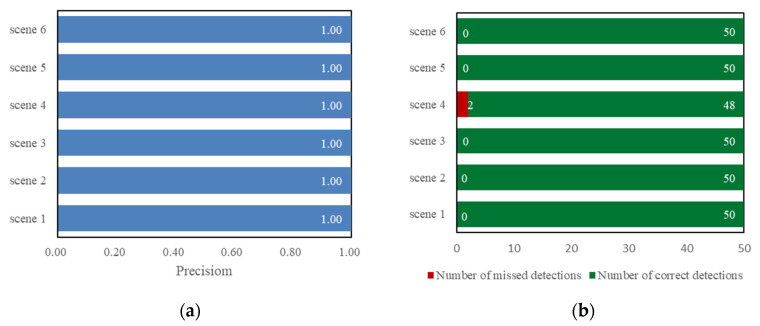
Water gauge detection model performance. (**a**) Detection precision; (**b**) Object missed detection. Scene 1, scene 2, scene 3, scene 4, scene 5, and scene 6 in the figure represent scenes with good daylight, infrared lighting at night, strong light, transparent water body, rainfall, and dirty water gauge, respectively.

The trained model is tested on the test set. During the test, the threshold of IoU [29] is set to 0.6. The test set consists of 300 positive sample images, divided into six groups in total, which are images in six scenes with good daylight, infrared lighting at night, strong light, transparent water body, rainfall, and dirty water gauge. The precision of the trained YOLOv5s on the test set and the number of missed samples are shown in Figure 9.

**Figure 10:**
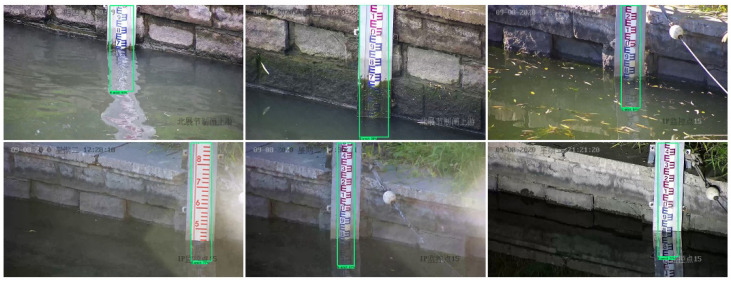
Water gauge detection effect.

It can be seen from Figure 9 that YOLOv5s has good precision for water gauge detection in different application scenarios, and has a faster detection speed, reaching 30 FPS. Each test image contains a water gauge object, and there is a big difference between the foreground part and the background part. The model has learned the characteristics of the water gauge well, and has a strong ability to check accuracy. As can be seen from Figure 9b, YOLOv5s shows better performance in scenes with good daylight, infrared lighting at night, strong light, rainfall, and dirty water gauge. However, in the transparent scene of the water body, there was a phenomenon of missed detection, and the water gauge part was mistakenly used as the background. The main reason is that when the water body is transparent, there is a reflection of the water gauge on the water surface and the saturation of the water surface part is low. The characteristics of the water gauge part and the water surface part are less different, and it is difficult to detect. The water gauge detection effect of YOLOv5s is shown in Figure 10 (In each sub-picture in the figure, the text in the upper left corner is the date the sample was collected, and the text in the lower right corner is the name of the video surveillance site).

### 4.3. Water Surface Line Recognition Performance

**Figure 11:**
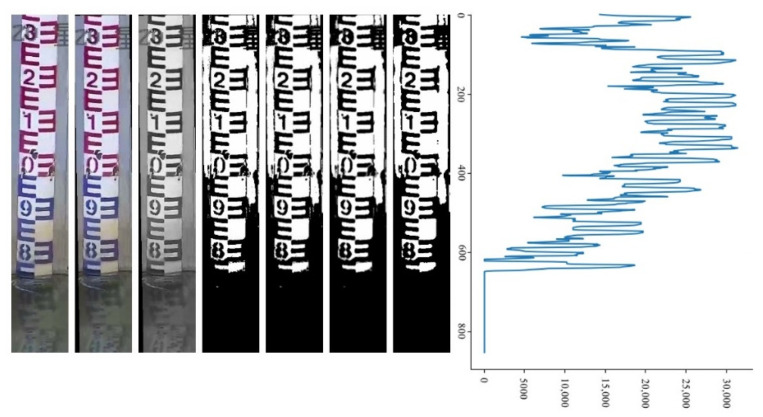
Classic effect of water level recognition.

Water surface line recognition is a critical step which directly affects the correctness of the water gauge reading. When detecting the water surface line, Hough transform, grayscale, denoising, and binarization are performed on the water gauge area detected by YOLOv5s to obtain a binary image, in which the water gauge appears as a black and white texture area. The classic effect of water level recognition is shown in Figure 11. The images from left to right are the original image, the tilt corrected image, the grayscale image, the binarized image, the Gaussian filtered image, the median filtered image, the binarized image, and the horizontal projection curve graph.

In order to detect the position of the water surface line, the pixel gray values in the binary image are accumulated row by row to obtain the horizontal projection curve. The horizontal projection curve is scanned from top to bottom. When a certain position is less than the set threshold δ for 10 consecutive times, the position is used as the row coordinate of the water surface line. Taking into account the interference of partial reflection on the water surface, small floating objects, after many experiments, we found that the water surface line is detected preferably when the threshold is 10% of the sum of the gray values of all white pixels in a row (δ = 255 × cols × 0.1).

This paper uses the image processing algorithms in the OpenCV library to carry out the water surface line recognition work. When the image is binarized, the threshold is set to 150. In this paper, a total of 600 water gauge images in six scenes with good daylight, infrared lighting at night, strong light, transparent water body, rainfall, and dirty water gauge have been tested for water surface line recognition. Among them, there are 100 images of each scene. Table 2 shows the number of samples in different error intervals of the water level recognition results.

It can be seen from Table 2 that under the conditions of good daylight, night infrared illumination, strong light, and rainfall, the water surface line detection effect is better. The proportion of samples with an error of less than 1 cm has reached 95%, and the speeds of detecting the water surface line in different scenes are not much different, all around 10 FPS. However, when the water body is transparent, the recognition error of the water surface line location increases; additionally, when the water gauge is seriously dirty, the detection effect of the water surface line is poor (the error size changes with the size of the dirty area). After analysis, the reason is that when the water body is transparent, the color characteristics of the water gauge and the water surface are not much different, the water surface is brighter, and the reflection of the water gauge is clearly visible on the water surface. As a result, binarization is difficult to distinguish the difference between the water gauge and the water surface. When the water gauge is seriously dirty, the water gauge part of the image after binarization is covered by a large number of black pixels, and the grayscale feature of the covered part is close to the water surface. During the binarization process, the covered part becomes black pixels, resulting in a large final water level data. In addition, under night infrared light conditions, the smooth water gauge surface reflects the light source to form a bright reflection on the water surface; because the grayscale is close to the water gauge background, it cannot be completely filtered out by binarization, and it appears as continuous small fluctuations in the horizontal projection. The results show that the continuous threshold judgment search strategy from top to bottom can effectively eliminate the influence of this local noise on water level recognition.

### 4.4. Scale Character Location and Recognition Performance

#### 4.4.1. Character Location Performance

Character location is also trained based on YOLOv5s; all settings are the same as described in Section 3.1, except that the dataset is different. Use the test set to test the trained model. The test set consists of 200 sample images, and each sample image contains multiple scale characters. The detection precision of the trained YOLOv5s on the test set and the number of missed objects are shown in Table 3.

It can be seen from Table 3 that the detection precision of the YOLOv5s is higher, reaching 99.7%, but there is a false detection phenomenon. The main reason is that the reflection of the scale character on the water surface is incorrectly detected as a scale character; the missed detection rate is low at 4.6%. What needs to be explained here is that because the proposed method is robust, the model only needs to detect at least one character, and the method is effective.

#### 4.4.2. Character Recognition Performance

**Figure 12:**
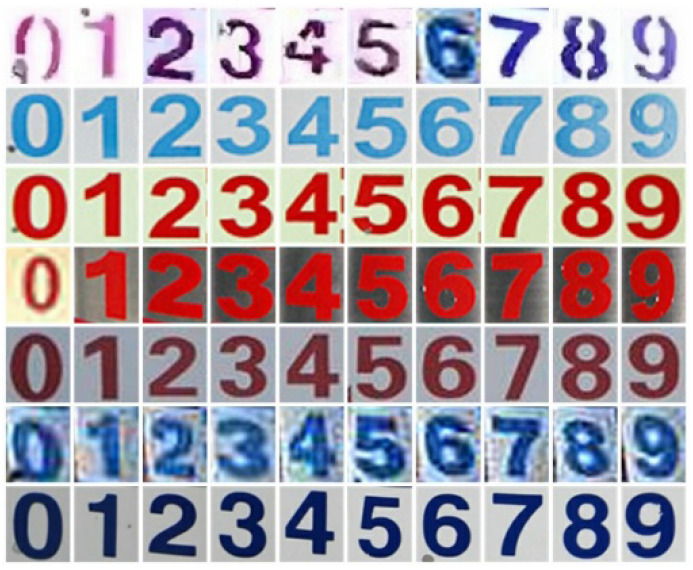
Sample images of the test set.

In the process of character recognition model training, the batch size is set to 96, the Adadelta optimizer is used for optimization, and the total number of iterations is set to 2000. In order to evaluate the performance of the scale character classification model of the water gauge, RGB color images from 0 to 9 categories were collected according to the color, posture, and clarity of the scale characters to form a test dataset with 50 images per category, for a total of 500 images (Figure 12).

The accuracy is used to evaluate the performance of the digital recognition model. The equation is:

(2) $$\mathrm{Accuracy}=\frac{\mathrm{TP}+\mathrm{TN}}{\mathrm{TP}+\mathrm{FN}+\mathrm{FP}+\mathrm{TN}}$$

The accuracies of the 10 categories of digital images are shown in Table 4.

By analyzing Table 4, it can be seen that the accuracy of the image classification model for each category of digital images is above 95%, and the average accuracy rate reaches 99.6%. It shows an excellent performance, a good generalization ability, and stronger stability. The character recognition model has reached the expected goal and can meet actual needs.

### 4.5. Performance Comparison of Water Gauge Reading

#### 4.5.1. Comparison Method

In order to verify the accuracy and reliability of the proposed method, it is compared with the traditional image processing method. We collected for comparison the images from a hydrological station in Beijing with good daylight, infrared lighting at night, strong light, transparent water body, rainfall, slightly dirty water gauges, and extreme scenes where water gauges are seriously dirty. Each scene is a group, and the first six groups each have 100 sample images. When the water gauge is dirty, it contains 50 slightly dirty sample images and 50 severely dirty sample images. A slightly dirty image shows that the water gauge has a little dirt, and a severely dirty image shows that the scale of the water gauge is completely covered by dirt.

#### 4.5.2. Performance Comparison

During the collection of image data, the water level of the hydrological station was stable, using an E-shaped vertical water gauge with a width of 20 cm and a 304 stainless steel material. The observation scene includes 6 common scenes, which are representative. The comparison results of the proposed method and water level acquisition method based on traditional image processing are shown in Table 5 and Table 6, respectively.

According to the results in Table 5, it can be seen that the proposed method works under good daytime illumination, nighttime infrared illumination, strong light illumination, and common rainfall scenarios, while the water level data error has reached more than 95% of the samples within 1 cm. Under normal lighting conditions during the day, the image is well lit, the object is clear, and it performs well in all steps of the automatic reading. Under night infrared lighting conditions, due to the better infrared reflection characteristics of the water gauge, the image object is clear, and the water level data obtained is more accurate. Under strong lighting conditions, due to the strong light, the discrimination between the object and the background is low and the strong light makes some parts of the object too bright; thus, the object contour and other identification information are lost, resulting in the increase of the number of samples with water level data error between 1–3 cm to 2. During the collection of sample images, the rainfall encountered was light rain. Under rainfall conditions, a lot of spot noises appeared in the sample images. However, it can be seen from the results that light rain has basically no effect on the acquisition of water level data. However, the performance is poor in the scenes of severely dirty water gauge and transparent water body, and there is a large error with the real water level data. In the water gauge stain test set, 50% of the water gauge is slightly dirty and 50% of the water gauge is heavily dirty. Slightly dirty water gauge has little effect on the water level acquisition data, but when the water gauge is seriously dirty, the dirt completely covers part of the water gauge, causing the water level data to be too large. If the heavily soiled part is removed, the average error of the system reaches 0.77 cm.

It can be seen from Table 6 that when the water level acquisition method based on image processing is well illuminated during the day, the number of samples with an error within 1 cm accounts for 56%, and the average error is 1.38 cm, which is the best in this scene. However, in other complex scene conditions, the water level recognition error is larger. It can be seen that this method is susceptible to complex environments. It should be pointed out that the camera angle of this method is fixed, and the angle cannot be switched freely. Due to the vibration of the camera caused by the wind, the error of the obtained data may increase, and even abnormal data may appear.

## 5. Conclusions

In this paper, we have proposed a novel water level gauge reading method based on machine vision. The proposed approach combines deep learning and image processing technology to significantly improve the accuracy and efficiency of water level monitoring. It was tested on a self-constructed dataset and compared with the traditional water level measurement method based on image processing. The results show that our proposed method can accurately automatically read the water gauge for most scenarios, with an error of less than 1 cm. Experiments have shown that YOLOv5s can accurately detect the water gauge in the video images and effectively eliminate the interference of complex backgrounds; when identifying the water level according to the horizontal projection of the binarized image of the water gauge area, the search strategy of continuous threshold determination can effectively eliminate the influence of local noises. Through YOLOv5s, all scale characters in the water gauge are detected, and the final water level elevation is calculated according to the pixel distance between the actual value represented by the detected lowermost scale character and the water level line. The strategy improves the versatility of the method. Compared with the traditional method based on image processing, the proposed method has the advantages of high precision, great stability, high efficiency, and continuous monitoring for 24 h. At the same time, due to the non-contact nature of the method, it can be applied in complex water environments, such as toxic, polluted, and turbid environments.

Although the proposed method shows good performance in most scenarios, our method still has certain limitations under extreme conditions. For example, when the part of the water level gauge close to the water surface is completely covered by silt or aquatic plants, the position of the water surface line obtained by the method in this paper is often higher, resulting in higher water level data.

As our future work, we will consider the use of deep learning to identify the position of the water surface to obtain further improvements. Potentially, this will make our method more robust. As another line of work, we plan to collect images containing water bodies to construct a training dataset. Based on this dataset, Mask R-CNN is trained. Then, Mask R-CNN is used to identify and segment the water body, and the intersection of the water body outline and the water gauge bounding box area is used as the position of the water surface line.

## Figures and Tables

**Table 1:** The structure of the character recognition model.

Layer	Kernel	Feature Maps	Activation Function
Conv layer	4 × 4	32	ReLU
Max-pooling	2 × 2	—	—
Conv layer	3 × 3	64	ReLU
Conv layer	3 × 3	64	ReLU
Max-pooling	2 × 2	—	—
Flatten layer	—	—	—
Dense layer	—	—	ReLU
Dropout layer	—	—	—
Dense layer	—	—	softmax

**Table 2:** Water surface line identification results.

Scene		Error ≤ 1 cm/Percentage	Error > 1 cm and Error ≤ 3 cm /Percentage	Error > 3 cm/Percentage	Speed (FPS)
Good daylight		95/95%	5/5%	0/0	11.3
Infrared lighting at night		100/100%	0/0	0/0	10.7
Strong light		99/99%	1/1%	0/0	11.4
Transparent water body		46/46%	45/45%	9/9%	11.2
Rainfall		95/95%	5/5%	0/0	11.4
Dirty water gauge	Slightly	49/98%	1/2%	0/0	8.5
	Severe	0/0	0/0	50/100%	8.4

**Table 3:** Test results of the scale characters.

Model	Precision	Number of Missed Objects/Percentage	Speed (FPS)
YOLOv5s	99.7%	46/4.6%	31

**Table 4:** Character classification results.

Class	Accuracy	Average Accuracy
0	100%	99.6%
1	100%	
2	100%	
3	98%	
4	100%	
5	100%	
6	100%	
7	100%	
8	98%	
9	100%	

**Table 5:** Results of automatic water level recognition by the proposed method.

Scene		Item					
		Processing Time (s)	Error ≤ 1 cm/Percentage	Error > 1 cm and Error ≤ 3 cm/Percentage	Error > 3 cm/Percentage	Average Error (cm)	
Daylight		0.34	95/95%	5/5%	0/0	0.57	0.77
Infrared Lighting at night		0.22	98/98%	2/2%	0/0	0.54	
Strong light		0.22	97/97%	2/2%	1/1%	0.63	
Transparent water body		0.68	45/45%	44/44%	11/11%	1.74	
Rainfall		0.16	91/91%	9/9%	0/0	0.64	
Dirty water gauge	Slightly	0.23	45/90%	5/10%	0/0	0.62	
	Severely	0.24	0/0	0/0	50/100%	5.37	—

**Table 6:** Results of the water level acquisition method based on traditional image processing.

Scene		Item					
		Processing Time (s)	Error ≤ 1 cm/Percentage	Error > 1 cm and Error ≤ 3 cm/Percentage	Error > 3 cm/Percentage	Average Error (cm)	
Daylight		1.50	56/56%	37/37%	7/7%	1.38	2.92
Infrared lighting at night		1.42	16/16%	75/75%	9/9%	1.97	
Strong light		1.51	43/43%	17/17%	40/40%	2.67	
Transparent water body		1.53	3/3%	5/5%	92/92%	5.37	
Rainfall		1.51	9/9%	9/9%	82/82%	4.72	
Dirty water gauge	Slightly	1.49	3/6%	30/60%	17/34%	1.44	
	Severely	1.55	0/0	0/0	50/100%	5.97	—

## Data Availability

Not applicable.
